# Complete Genome Sequence of the Predatory Bacterium *Ensifer adhaerens* Casida A

**DOI:** 10.1128/genomeA.01344-17

**Published:** 2017-11-22

**Authors:** Laura E. Williams, David A. Baltrus, Sean D. O’Donnell, Tara J. Skelly, Mark O. Martin

**Affiliations:** a Department of Biology, Providence College, Providence, Rhode Island, USA; b School of Plant Sciences, University of Arizona, Tucson, Arizona, USA; c School of Animal and Comparative Biomedical Sciences, University of Arizona, Tucson, Arizona, USA; d Department of Genetics, University of North Carolina at Chapel Hill, Chapel Hill, North Carolina, USA; e Biology Department, University of Puget Sound, Tacoma, Washington, USA

## Abstract

We report here the complete genome sequence of the facultative predatory bacterium *Ensifer adhaerens* strain Casida A. The genome was assembled into three circular contigs, with a main chromosome as well as two large secondary replicons, that totaled 7,267,502 bp with 6,641 predicted open reading frames.

## GENOME ANNOUNCEMENT

*Ensifer adhaerens* strain Casida A was first isolated in 1980, when Pennsylvania soil samples were enriched for microbes that could prey upon *Micrococcus luteus* (1), and described in greater detail in 1982 (2). *E. adhaerens* represents an isolate with unique biological capabilities in that it is a facultative predator that can target some Gram-positive strains, such as those belonging to the genus *Micrococcus* (3). To date, little is known about the genetics of predation in *Ensifer* strains or which pathways are required for these strains to track *M. luteus*, but a complete genome sequence provides a first step toward identifying pathways of interest in this unique predatory bacterium.

Genomic DNA for PacBio and Illumina libraries was prepared from populations initiated with single colonies and purified as per Baltrus et al. (4). Genomic DNA was independently extracted for the creation of each library type. PacBio sequencing was carried out at the University of North Carolina (UNC) Genomics Core facility on an RSII instrument over four separate single-molecule real-time (SMRT) cells using P6-C4 chemistry. There were 601,168 total polymerase reads across all SMRT cells. Illumina sequencing was performed as part of one multiplexed lane on an Illumina HiSeq platform with 100-bp paired-end libraries at the University of Arizona Genomics Core facility. There were 7,971,492 total reads in the Illumina library, yielding approximately 100× coverage of the genome.

Assembly of PacBio data was performed in an Amazon EC2 instance of SMRTPortal using Hierarchical Genome Assembly Process (HGAP3) (5) with default parameters and an expected genome size of 7.3 Mb. Subread filtering yielded 124,810 subreads with a mean length of 6,143 and an *N*_50_ of 8,669. Assembly of these subreads generated three large contigs of 4,071,185 bp (93× coverage, 63% GC content), 1,736,943 bp (pCasidaAA, 63% GC, 77× coverage), and 1,459,374 bp (pCasidaAB, 60% GC, 76× coverage). The total published assembly size was 7,267,502 bp. This number of replicons and nucleotide content is consistent with what is found in genomes of strains closely related to *Ensifer adhaerens*, with one main chromosome and two large secondary replicons. Illumina reads, trimmed for quality, were then used to manually correct the PacBio library. Briefly, Illumina reads were mapped to each large contig from the Hierarchical Genome Assembly Process (HGAP) assembly, and single nucleotide variants were mapped using Geneious version 6.0.5 with default parameters. Variants in the PacBio assembly supported by at least 90% of Illumina reads (with a minimum read depth of 10×) were then manually corrected for the final assembly.

The genome of *E. adhaerens* strain Casida A was annotated using the NCBI Prokaryotic Genome Annotation Pipeline (6) and is predicted to code a total of 6,653 open reading frames (ORFs). The main chromosome contains 3,849 predicted ORFs, 9 rRNAs, 55 tRNAs, and 45 potential pseudogenes. The next largest replicon contains 1,513 predicted ORFs, 3 rRNAs, 3 tRNAs, and 47 potential pseudogenes. The smallest replicon contains 1,279 predicted ORFs, 3 rRNAs, 4 tRNAs, and 95 potential pseudogenes.

### Accession number(s).

This whole-genome shotgun project has been deposited at GenBank under the accession no. CP015880 (chromosome), CP015881 (plasmid pCasidaAA), and CP015882 (plasmid pCasidaAB). The versions described in this paper are the first versions, CP015880.1, CP015881.1, and CP015882.1, respectively.
